# Stroke Lesion Segmentation and Deep Learning: A Comprehensive Review

**DOI:** 10.3390/bioengineering11010086

**Published:** 2024-01-17

**Authors:** Mishaim Malik, Benjamin Chong, Justin Fernandez, Vickie Shim, Nikola Kirilov Kasabov, Alan Wang

**Affiliations:** 1 Auckland Bioengineering Institute, The University of Auckland, Auckland 1010, New Zealand; mmal151@aucklanduni.ac.nz (M.M.); ben.chong@auckland.ac.nz (B.C.); nkasabov@aut.ac.nz (N.K.K.); 2 Faculty of Medical and Health Sciences, The University of Auckland, Auckland 1010, New Zealand; 3 Centre for Brain Research, The University of Auckland, Auckland 1010, New Zealand; 4 Mātai Medical Research Institute, Gisborne 4010, New Zealand; 5 Knowledge Engineering and Discovery Research Innovation, School of Engineering, Computer and Mathematical Sciences, Auckland University of Technology, Auckland 1010, New Zealand; 6 Institute for Information and Communication Technologies, Bulgarian Academy of Sciences, 1113 Sofia, Bulgaria; 7 Knowledge Engineering Consulting Ltd., Auckland 1071, New Zealand; 8 Medical Imaging Research Centre, The University of Auckland, Auckland 1010, New Zealand; 9 Centre for Co-Created Ageing Research, The University of Auckland, Auckland 1010, New Zealand

**Keywords:** stroke, lesion segmentation, deep learning, network

## Abstract

Stroke is a medical condition that affects around 15 million people annually. Patients and their families can face severe financial and emotional challenges as it can cause motor, speech, cognitive, and emotional impairments. Stroke lesion segmentation identifies the stroke lesion visually while providing useful anatomical information. Though different computer-aided software are available for manual segmentation, state-of-the-art deep learning makes the job much easier. This review paper explores the different deep-learning-based lesion segmentation models and the impact of different pre-processing techniques on their performance. It aims to provide a comprehensive overview of the state-of-the-art models and aims to guide future research and contribute to the development of more robust and effective stroke lesion segmentation models.

## 1. Introduction

Stroke is currently the second leading cause of death and the third leading cause of disability worldwide [1]. As per the World Stroke Organization, around 15 million people suffer from stroke annually; out of these 15 million, about 43% lose their lives, and of the survivors, roughly two-thirds have some disability [2]. Currently, only qualitative lesion assessment is a part of the clinical workflow, complimented by various assessments such as the National Institutes of Health Stroke Scale [3] and the Cincinnati Prehospital Stroke Severity Scale [4] to gauge stroke severity. Different medical imaging modalities, such as non-contrast computed tomography (CT) and magnetic resonance imaging (MRI), can support the subjective assessment. Both modalities have pros and cons, briefly discussed in Table 1.

In the last decade, machine and deep learning development has grown exponentially. Artificial intelligence is now being used in every field of life to reduce human effort, and medical image analysis is no different. Deep learning methods are used for automated stroke classification and rehabilitation prediction [5]. However, at the heart of these processes is lesion segmentation, a term used to define the process of tracing lesion outlines by categorising each voxel as a lesion or non-lesion in a medical image [6]. Segmented lesions can allow the model to learn the anatomical features and their impact on the prediction. The gold standard for lesion segmentation is manual segmentation [7]. Computer-aided diagnostics tools and techniques, such as 3D Slicer [8], ITK-SNAP [9], Amira [10], and MIPAV [11], are some of the open-source and commercially available tools that are helpful for lesion segmentation, analysis, and visualisation. However, some human intervention and verification is still required. It is also time-consuming and laborious to segment the lesions layer by layer [12], and the chances of error and bias are also high as lesions could be of irregular size and shape in each layer [7,13], which necessitates the development of automated techniques.

Different deep learning techniques, such as convolutional neural networks (CNNs) and transformers, are employed to perform lesion segmentation. The primary objective of this review is to critically analyse existing research on deep-learning-based lesion segmentation in stroke analysis, providing a comprehensive guide for future research directions. We do not present new findings but investigate the usage of the different deep learning models within the current literature and identify promising research avenues. We also aim to investigate the usage of different pre-processing techniques within the literature and their impact on the model’s performance.

Different databases and platforms were used to gather the literature for this study, including Google Scholar, PubMed, arxiv.org, ScienceDirect, and SpringerLink. Terms such as “Stroke Lesion Segmentation”, “Deep Learning”, “Ischaemic Stroke”, and “Neural Network” were used to search for the literature. Since much work has been carried out in deep learning and lesion segmentation, this study limited its search to between 2018 and 2023 to focus solely on the latest state-of-the-art studies. The recently introduced Must AI Criteria-10 (MAIC-10) [14] checklist was used to evaluate the quality of technical studies. The Supplementary Materials Table S1 provides further details on the scoring of each chosen study.

**Table 1:** A brief side-by-side comparison of MRI and CT.

Aspect	MRI	CT
Time Constraints	An MRI scan may require up to an hour to conclude its findings [15]. However, some medical centres have reduced the time to up to 10 min using different protocols [16].	A CT typically takes between 5 and 15 min per scan.
Cost Effective	MRI costs almost double compared to a CT [17].	A CT costs half the amount of an MRI [17].
Ischaemic Lesion Detection	Since MRI scans produce detailed images, detecting small lesions is easier [18].	CT scans are good at detecting large ischaemic lesions. It might be challenging to catch a small lesion earlier using a CT scan [18].
Haemorrhage Detection	MRI scans are suitable for detecting small or chronic haemorrhages [18].	CT scans perform well while detecting acute or larger haemorrhages. [19].
Lesion Visibility	As an MRI produces a more detailed image, it is easier to detect and visualise a lesion. A lesion is more evident in the hyperintense region using a DWI map [20].	Due to low contrast, a lesion is harder to visualise in a CT scan [20].
Easier segmentation	It is easier to segment a lesion using an MRI scan manually. The different modalities, such as DWI, FLAIR, and T2-weighted, can be used to perform segmentation more accurately [21].	Due to the low tissue contrast, it is harder to manually segment a lesion using a CT scan [21].
Health Concerns	The magnetic rays emitted by the MRI scanner can disrupt the working of different implanted devices.	Since CT scanners use ionising radiation, they can cause cellular damage.

## 2. Previous Literature Surveys

Since the early 2000s, considerable efforts have been made towards automating lesion segmentation and amalgamating the findings of technical studies in the form of literature and systematic surveys. With the boom of machine learning techniques in lesion segmentation, researchers have attempted to present current trends concisely. For example, Rani et al. [22] discussed the various object recognition and localisation techniques that can be applied to lesion segmentation. They included widely used techniques like region-based convolutional neural networks [23], You Only Look Once [24], Single Shot MultiBox Detector [25], and EfficientNet [26] in their discussion. One of the highlights of this study was that the authors also compared the algorithms in terms of speed and performance.

Karthik et al. [27] discussed in detail various studies that used machine and deep learning methods. The authors of this study did not limit themselves to stroke segmentation; they also expanded the study to discuss machine learning techniques for stroke classification. The study concluded that using smaller datasets made many analysed models prone to overfitting and class imbalance. To remedy the presented issues, the possible solutions given by the authors included the usage of data augmentation or employing generative adversarial networks (GANs).

Both studies, one conducted by Thiyagarajan and Murugan [28] and the other by Zhang et al. [6], presented a very in-depth analysis of the latest deep learning techniques, where Zhang et al. [6] broke down the techniques based on the dataset they used, and Thiyagarajan and Murugan [28] presented a step by step analysis of each study under discussion. Zhang et al. [6] presented a conclusion similar to the one given by Karthik et al. [27]; they compared the different factors affecting the performance of stroke lesion segmentation to brain tumour segmentation. They concluded that using GANs and employing different augmentation techniques can help with the problem of smaller datasets. Another solution presented was using features gathered from the MRI alongside the doctor’s expertise in stroke treatment as supplementary input factors for better information extraction from the image. The authors also discussed the advantages and disadvantages of using deeper or shallower models while segmenting smaller lesions. Thiyagarajan and Murugan [28] concluded that DWI modalities present overall better segmentation results for acute and sub-acute stroke, whereas T2-weighted and FLAIR perform better for chronic stroke.

Karthik and Menaka [29] discussed pre-processing techniques, lesion segmentation, and classification techniques. Their review focused on manual and deep learning segmentation techniques for lesion segmentation. Karthik and Menaka [29] concluded that segmentation techniques could be improved by incorporating structural and symmetrical properties of lesions into the models.

Wang et al. [30] discussed some state-of-the-art lesion segmentation techniques. They concluded that multi-centre data might be required to improve the performance of the AI-based models. They also presented the idea of using CT images as input as they are commonly used in clinical practice rather than MRI. Abbasi et al. [31] presented a thorough analysis of current deep learning models. They compared the different models based on image modalities, i.e., CT and MRI. Abbasi et al. [31] also presented a similar conclusion to previous studies that data augmentation techniques must be explored for better results. They also concluded that integrating multi-model imaging modalities can help better understand ischaemic stroke and improve the segmentation results.

Table 2 presents the contributions of the review papers discussed above.

## 3. Stroke Lesion Segmentation

**Figure 1:**
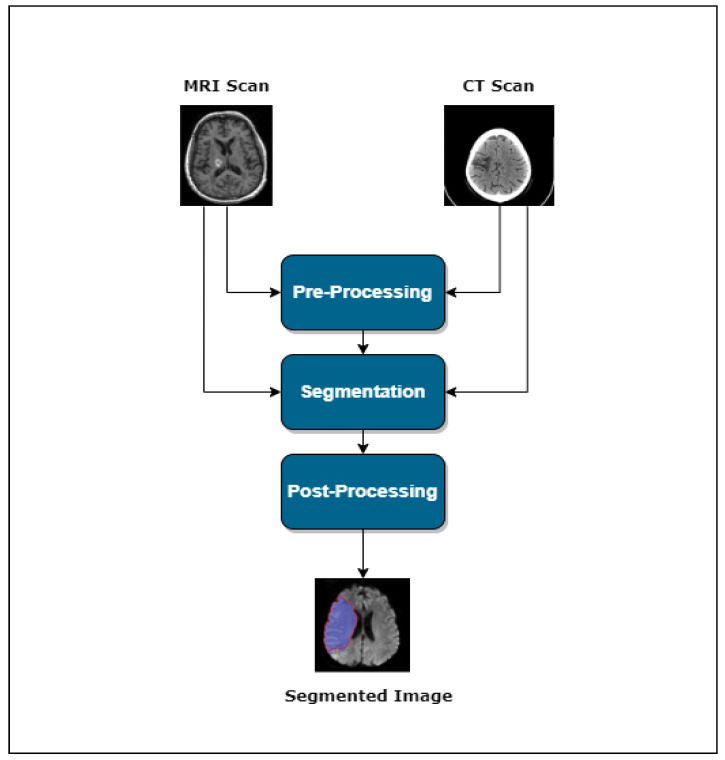
Lesion segmentation pipeline.

Usually, one can divide the lesion segmentation model into three main modules. Figure 1 shows an overview of the process.

1. Pre-processing
2. Segmentation
3. Post-processing

This section provides a basic overview of the different techniques used in the literature for pre-processing and segmentation.

### 3.1. The Role of Pre-Processing in Stroke Lesion Segmentation

Pre-processing is the first step in solving any computer vision problem, and stroke lesion segmentation is no different. The purpose of adding a pre-processing layer is to remove any noise added to the image during the image acquisition phase. As deep learning models often require a considerable amount of data, the data are often collected from multiple centres. The acquisition equipment and methodology difference can often introduce inter-site and intra-site variabilities in the data [32]. The presence of these variabilities might lead to the introduction of noise and bias in the data, which can negatively impact stroke lesion segmentation. Pre-processing is crucial for accurate stroke lesion segmentation, removing noise and bias introduced by multi-centre data collection and preparing data for deep learning models despite the trend towards end-to-end learning. Hence, some studies omit the pre-processing stage and input the data directly to the network, as seen in [6,13] among others.

However, within our literature, we observed that studies still use pre-processing techniques; for example, Clèrigues et al. [33] used symmetric modality augmentation, which allowed them to learn features based on the symmetry of the brain hemisphere using image registration. Soltanpour et al. [34] applied multiple techniques to clean their data, such as intensity clipping, to ensure no high-intensity outliers were present. They also employed image registration using bilinear interpolation to replace each value from their sample image and its corresponding ground truth with the weighted average of their 2 × 2 neighbour mean. Sheng et al. [35] also used bilinear interpolation.

Image registration techniques establish a point-by-point correspondence between anatomical features present in two scans of the same organ taken from different angles [36]. This technique is beneficial as different modalities and angles are available per scan for medical images, all of which have different information. With the increased data, capturing the spatial information, such as the anatomical structures, in the scans is essential. It can be performed manually using cross-correlation-based, Fourier-transformation-based, landmark-based mapping methods [37] or by using machine learning techniques [38,39]. Within our literature, Hui et al. [40], Liu et al. [41], Wu et al. [42] applied image registration to its input data by transforming each image to MNI-152 space.

Skull stripping removes the bone structure from a medical image to ensure that only brain tissue is considered during segmentation [43]. Different methods of performing skull stripping include morphology-based [44,45], intensity-based [46,47], and atlas-based [48] methods. Traditional skull stripping methods are sensitive to noise [49,50]; hence, machine-learning-based skull stripping methods have also been introduced to cater for the sensitivity to noise [51]. Within our literature, skull stripping was applied by Anand et al. [52], Cui et al. [53], Karthik et al. [54] to remove all non-brain tissue, including the blood vessels, fat, muscles, and dura mater, making the lesion visibility better within the medical image.

Another pre-processing method favoured by studies such as Hui et al. [40], Ahmad et al. [55], Tureckova and Rodríguez-Sánchez [56] is bias correction, which removes nonuniformity from the input images. The data can introduce nonuniformity due to variations in equipment quality and spatial inhomogeneity caused by magnetic waves or X-rays [57,58]. Bias correction can be performed using multiple techniques, including filtering, surface fitting, and histogram-based techniques [59].

Though many studies favour bias correction and skull stripping, they are often followed by or used with normalisation. Normalising an image allows the model to generate a more stable output. It can be performed by changing the intensity of each pixel value to resemble the characteristics of pre-defined data [60]. The least square method, radiometric normalisation, and histogram normalisation are some of the most commonly used normalisation techniques [61]; machine learning has also been introduced for image normalisation [62,63]. Within our literature, it was observed that z-score normalisation, also called standardisation, is the most common choice, as it was used by [34,52,53,54,56,64]. Other techniques employed for normalisation include min-max normalisation used by Dolz et al. [65], and percentile clipping favoured by Wang et al. [66].

### 3.2. Advancements and Diverse Architectures in Automated Lesion Segmentation

The introduction of convolutional neural networks revolutionized the field of machine learning; with their inherent ability to learn features directly from the input image, they became the most commonly used machine learning technique for computer vision problems. This technique is also commonly used for lesion segmentation, especially after the U-Net [67] model was presented. GAN is another technique employed for lesion segmentation tasks; it is beneficial for low-resource models and unsupervised machine learning approaches.

#### 3.2.1. Supervised Learning

*Advancements and Variations OF U-Net Architecture*

During the literature-gathering process, it was observed that many of the state-of-the-art techniques stem from the U-Net model. Some of them focused on improving the current structure, while others used the model as a base for their own. Clèrigues et al. [33] introduced residual connections in the basic U-Net model and replaced the commonly used ReLU function with PReLU; Dolz et al. [65] presented the idea of using multiple encoders for different modalities of the scan, each of which was densely connected with others for feature preservation. Karthik et al. [54] focused on how different loss, activation, and optimization functions can impact the U-Net model. They tested the model’s performance by using different combinations of these functions. Hui et al. [40] used two U-Net structures rather than one to capture the auxiliary and primary features separately. The two networks are identical, except they were trained using different loss functions.

Ou et al. [68] proposed a version of U-Net consisting of lambda layers [69] rather than convolution layers. The concept behind lambda layers is to capture the context of each value by converting it into a linear function referred to as a ”lambda”. The introduced model calculates global, local, and inter-slice lambda from a 3D feature map. All three lambdas are applied to the query, the pixel under study, to produce the final results.

Soltanpour et al. [34] presented two enhancements on a U-Net-based model called MultiResUNet [70]. Firstly, they used different filter sizes than the original model’s 3 × 3 filter. They replaced skip connections between each layer with CNN-based shortcuts where each shortcut block consisted of four 3 × 3 convolution layers, whose results were concatenated with a 1 × 1 convolution layer. The first enhancement allows the model to capture features on multiple scales, whereas the second change balances out the original model’s semantic gap. Sheng et al. [35] introduced a block called the Cross-Spatial Attention Module, which is used instead of the skip connection. Using this block rather than simple skip connections enables the feature map to capture the spatial information more accurately.

Liu et al. [71] proposed a model incorporating dense blocks instead of simple convolutions in the encoder; they also used two side-by-side encoding and decoding structures to capture all the features. Ahmad et al. [55] also presented a dense block-based structure, with a residual inception block added after the first convolution layer and used as the bottleneck layer; they also employed a deep supervision technique in the decoder for better convergence. The dense block presented in this study consisted of three convolution layers, with the first convolution layer followed by a pooling layer, whereas Liu et al. [71] followed the structure of DenseNet-121 but extended to 123 layers.

A dense block consists of multiple convolutional layers directly connected with each subsequent layer; this structure allows each layer to reuse the features from all previous layers and avoid overfitting [72]. Within the scope of the above studies, it was observed that Liu et al. [71] did not present a significant improvement in the results; however, it was the opposite case for Ahmad et al. [55] who presented a significant improvement from the baseline models. It should be noted that Ahmad et al. [55] used dense blocks combined with residual inception blocks within their decoder, which could factor in the improved results.

Tureckova and Rodríguez-Sánchez [56] experimented by replacing the convolution layers with dilated ones; they experimented by placing the dilated layers in different network modules and concluded that dilated filters in the first convolution layer produced the best results. Omarov et al. [73] also presented similar work; they introduced different optimization layers in the original U-Net model and used dilated convolutions in some layers to increase the filter’s field of view. Using dilated filters instead of simple filters allowed both studies to increase their field of view without increasing the filter size or computational power.

Liu et al. [41] presented a Multi-scale Deep Fusion unit in the bottleneck layer of U-Net; the authors employed the techniques of Atrous Spatial Pyramid Pooling (ASPP) [74] and capsules with dynamic routing [75] to capture and encode the global context. Zhang et al. [12] also employed the concept of ASPP in their bottleneck layer. The ASPP produced a fused feature map using the features produced from U-Net’s encoding layer and the feature set produced by the residual encoder. The residual encoder used multiple convolution layers to capture the low-level features. Similarly, the work proposed by Zhou et al. [64] used 2D and 3D convolutions in the encoder to maximize the information. A dimension transformation block was used in the bottleneck layer to control the number of trainable parameters. Qi et al. [76] used a feature similarity module in the bottleneck layer. They also replaced the convolution layers of the U-Net model with their proposed block called the X-block. The X-block used depth-wise convolutions to capture the contextual information present in the input scan. Using depth-wise convolutions allowed the models to perform similarly to the baseline models but with almost half the trainable parameters.

*Evolution towards Capturing Global Features*

The previously discussed studies focused on capturing the local features present in the image. However, some recent studies also showed the importance of capturing global features. Since every lesion is variable, capturing the contextual and spatial information present in the scan is crucial. A CNN-based model captures local features well, whereas transformers perform competently at capturing global features.

Wu et al. [42] employed both techniques in a ‘W’-like structure to capture the local and global features present in the input image. They introduced two new modules, the Boundary Deformation Module (BDM) and the Boundary Constraint Module (BCM). The proposed model first uses a U-Net to capture the local features; the calculated features are then processed through the BDM module before passing them to the transformer’s encoder. The transformer’s decoder comprises a similar model to the U-Net’s decoder; it consists of different up-scaling convolution layers, and the output of these layers is processed through the BCM layer to ensure the pixels neglected in the previous layers can also contribute to the final segmentation. The BCM uses different dilated convolution layers to capture the context of the overall image.

Wu et al. [77] proposed a similar technique where a transformer layer was introduced in the encoder network to capture the long-range relationships between the features. The authors proposed a model called MLiRA-Net, comprising a patch partition block (PPB), the MLiRA mechanism, which is the unit used for feature extraction and is called the multi-scale long-range interactive and regional attention mechanism, and lastly, the feature interpolation path (FIP), which acts as the decoder. The PPB consists of two cascading convolutional layers; it captures the local features present in each input image patch. The FIP module consists of multiple combinations of convolutional and transpose convolutional layers, each connected to its relevant encoder layer using a skip connection.

*Feature Extraction Variations*

Most models discussed above had an end-to-end structure, with the feature extractor embedded in the proposed model. However, some works used a separate feature extraction module, such as Wang et al. [66] who proposed a model that used a CNN-based feature extractor that captured low-level and high-level features from CT perfusion maps; these features were then used to generate a pseudo-DWI image, which was then encoded and later on decoded to extract the segmentation. The U-Net model was the base model for segmentation, with the batch normalization layers replaced with switchable normalization and a Squeeze-and-Excitation block to capture the channel-wide features.

*Beyond U-Net: Novel Segmentation Techniques*

Even though the U-Net structure has been heavily utilized, not all studies used it. Karthik et al. [78] proposed a fully connected CCN-based network. The network consisted of multiple layers of the Multi-Residual Attention Block, which processed mainline and input context features using multiple convolutional and down-sampling layers. The authors also proposed a masked dropout module, which was applied only on the produced mainline features to regulate the receptive field. The decoder model combines low- and high-level features with skip connections from each intermediate encoder layer. The final results are produced by integrating features of the different receptive fields. Unlike the U-Net model, the layers of the decoder work independently of each other, and majority class voting is used to produce the segmentation result. Li [13] presented a CNN-based model inspired by the human visual cortex. The proposed model consisted of three blocks called V1, V2, and V4, respectively, with a bottleneck layer. Each block of the model has a unique structure and is used to capture a specific type of information.

Liu et al. [79] proposed an encoder/decoder-based model comprising a ResNet and a global convolution network (GCN)-based encoder/decoder structure. The authors used their internal dataset, comprising MRI scans of 212 ischaemic stroke patients; DWI, ADC, and T2-weighted modalities were generated for each patient. Each modality was concatenated to a three-channel image, which acted as the input for the model. Each input image passes through a series of Res-Blocks where each block can contain an n-number of bottleneck layers. The output of each Res-Block is passed to its corresponding up-sampling layer using a skip connection comprising of a GCN and Boundary Refinement layer before it is passed to the next block. Anand et al. [52] also employed an encoder/decoder-based model; the encoder used multiple DenseNet-based layers. The input image comprises five channels, one for each modality of CT, including CBV, CBF, MTT, and $T_{max}$.

Ou et al. [80] presented a transformer-based encoder/decoder structure called a Patcher. The encoder consisted of multiple cascading layers of patcher blocks, where each patcher block uses the combination of CNN and vision-based transformers to capture both local and global features in the image. The decoder follows the idea of the mixture of experts (MoE) technique. Firstly, multi-layer perceptrons (MLP) are used to process the feature maps produced by each encoding layer. All weights are then up-sampled and concatenated to be processed through another MLP to produce the final prediction. Though the model outperformed all baseline models, one drawback of using a vision transformer-based model would be that it is currently only limited to 2D data.

#### 3.2.2. Semi-Supervised Learning

Accurately predicting stroke boundaries is one of the primary goals of automating stroke lesion segmentation. However, stroke lesions are often very irregular in shape, size, and form, making them harder to generalize without a large corpus of data. Though the different variants of the ATLAS [81,82] and ISLES [83,84] datasets provide various types of annotated stroke data, the models trained on them still need help with unseen data. Some studies have employed semi-supervised techniques to improve the generalization of the model. For example, Cui et al. [53] introduced a DeepMedic [85]-based student–teacher model. Both models use the same structure, and their weights are updated alternatively. The teacher model is initialized with the DeepMedic model, whereas the student model learns by minimizing the loss of the teacher model.

Another approach was presented by Zhao et al. [86], which used weakly labelled data in combination with fully labelled data in their presented model. The model comprises three modules: classification, segmentation, and inference. The VGG16-based classification module was trained on weakly labelled data and generated class activation mappings (CAM) for the inference network and a feature map for the classification network. The inference module, finally, uses the binarized segmentation produced from the segmentation model and the CAM produced from the classification module to produce the prediction.

#### 3.2.3. Unsupervised Learning

Deep learning models require a lot of labelled data to learn real-world patterns accurately; however, labelled medical imaging data are scarce for various reasons, including but not limited to privacy issues and domain expertise required to label the data [87]. Hence, lesion segmentation has expanded to unsupervised techniques, predominantly using GANs. Each GAN comprises two modules: the generator and the discriminator. The generator generates data sample points, and the discriminator needs to determine whether the samples were from real-world data or were generated by the generator. As it is in the model’s name, both the discriminator and generator are trained in an adversarial manner, where the generator improves its ability to generate real-world data, and the discriminator improves its differentiation abilities [88].

As discussed in the preceding sections, recent research shows a prevalent use of the U-Net model. Even in unsupervised learning, most studies employ U-Net, or one of its variants, as their generator for GANs. For instance, Islam et al. [89] used a U-Net-based segmentation/generator model within the GAN framework. However, the structure of the discriminator varies from study to study, continuing with the example of Islam et al. They used an FCN-based discriminator consisting of four 2D convolutional layers followed by ReLU activation.

Another notable approach was used by Ou et al. [90]; they used a U-Net-inspired transformer model discussed beforehand called Patcher [80] as their segmentation module and used an FCN-based discriminator. Wang et al. [91] proposed an unsupervised approach called a consistent perception generative adversarial network. The proposed GAN comprises a segmentation, an assistant, and a discriminator model. The segmentation model uses the structure of U-Net in combination with a similarity connection module [76]. The assistant model assists the discriminator in assessing whether the input image is fake or the target image.

## 4. Results and Future Directions

Table 3 summarises the studies considered for this review, offering insights into the state-of-the-art stroke lesion segmentation techniques.

Of the chosen studies, 71% used MRI as their input modality, and 29% used CT. T1-weighted images were the preferred choice for MRI, followed closely by DWI. However, using the modalities in combination was the preferred option for CT. Most studies under consideration used supervised learning, with 53% having a U-Net-based structure. Binary cross-entropy loss and dice loss, or using them in combination with other losses, was the most common choice among the studies.

### 4.1. Data Dimensionality and Its Processing Techniques

Two-dimensional data or images were used by 71% of the studies as input for their model, whereas 29% used 3D data. A common trend in the studies that used 3D data was to down-sample the input. The down-sampling was performed due to the memory limitations of the GPUs. As well as down-sampling, patching was a technique that was applied to the data as shown by Qi et al. [76] and Wang et al. [91].

Future models using 3D data can explore patching further as they are less memory extensive and do not lose data like down-sampling. An added advantage of patching is that it can help cater to the class imbalance problem. In stroke data, the healthy tissue is far greater in number than the lesion, which causes the class imbalance problem, which often leads to underfitting of the model; sophisticated patching techniques can help cater for this problem as well.

### 4.2. Data Pre-Processing

Normalization was the most commonly used pre-processing technique, followed by skull stripping and bias correction. Of the 28 studies chosen, 46% did not mention using pre-processing techniques; however, it should be noted that they all used public datasets, which were pre-processed beforehand to normalize the data [81,82]. Of the studies that mentioned pre-processing techniques, 46% used normalization, 10% used skull stripping, and 7% used bias correction.

Since most studies used 2D data, reviewing more sophisticated pre-processing techniques, such as harmonization and super-resolution, is suggested as a future research avenue. Image harmonization normalizes and standardizes the data collected from different sources [92]. Studies have employed convolutional neural networks, transformers [93], and attention modules [94] for image harmonization. Super-resolution-based techniques improve the captured image’s resolution or quality.

Though these techniques may add a layer of complexity to the model, they can make the data more centralized and make it easier for the model to see the patterns.

### 4.3. Data Augmentation Trends

Another trend noted during the review was the use of data augmentation. Data augmentation using flipping, rotating, and rescaling of the image was used by 45% of the studies using 2D data—however, none of the techniques using 3D data applied augmentation. In the future, data augmentation for 3D data could also be employed; as demonstrated by Cirillo et al. [95], 3D augmentation may increase the effectiveness of the segmentation model. GANs [96,97], multi-planar image synthesis [98], and affine transformations [99] are some of the techniques that can be applied to 3D data augmentation [100]. Though it should be noted that 3D data are already memory-extensive, adding more 3D volumes could increase the training time of the models extensively.

### 4.4. Enhancing Segmentation Using Transfer Learning

Transfer learning is a widespread technique employed for training different machine learning models; it uses previous experiments’ knowledge to fine-tune the current problem’s features. This technique is particularly beneficial for medical information or image analysis, as the data are limited for various reasons, including the disease’s rarity and ethical and legal issues [59].

As mentioned, most studies used the U-Net or the encoder/decoder model as a base model; hence, the weights trained in a previous study can be used as a starting point rather than training the model from scratch [101]. From our current literature, only three studies employed transfer learning. ImageNet [102,103] was the standard choice for model initialization between all three studies. Wu et al. [77] used weights pre-trained on the ImageNet model to initialize their transformer layers. Anand et al. [52] used the weights to initialize their DenseNet-based encoder layer, and Zhao et al. [86] used them to initialize their VGG-16-based model.

However, it should be noted that the ImageNet model contains coloured real-world images. In contrast, the medical images are primarily in greyscale and are much noisier than natural real-world images [104]. Hence, a more appropriate choice of transfer learning could be using medical image-based datasets, such as the newly introduced RadImageNet [105], which includes weakly labelled medical images of different organs. Another technique would be to use models trained on brain tumour data, as carried out by [106]. These techniques can potentially increase the effectiveness of transfer learning in stroke lesion segmentation as they are tailored for medical images.

## 5. Limitations

This study has several limitations. Firstly, it only incorporated technical studies presented in English that were considered for the review. As a result, the findings may offer a partial landscape for research being conducted in lesion segmentation. Secondly, the survey was performed using a particular set of keywords. Any technical study whose title or abstract did not contain the keywords was also not considered for the study. Lastly, the selected studies only included technical studies that focused solely on lesion segmentation; techniques presented in combination with stroke classification and rehabilitation were also not considered. Hence, it narrows the scope of the study and limits it from providing a broader context about stroke lesions.

## 6. Conclusions

In this review, we examined the different state-of-the-art machine learning models alongside different strategies and techniques currently being utilised to improve the performance of these structures. It can be easily concluded that the medical image analysis landscape evolved significantly after the introduction of deep learning techniques. U-Net and transformer-based structures have shown great promise in accurately identifying and segmenting stroke lesions in both MRI and CT.

Since there is a lack of substantial multi-centre-based data to train deep learning models, different augmentation and pre-processing techniques were applied to increase the effectiveness of the models. However, there is a need to analyse advanced pre-processing techniques in conjunction with lesion segmentation. Additionally, improving the utilisation of transfer learning is crucial.

While this paper reviews methods for the analysis of neuroimaging data of stroke patients, it will be interesting to see how these methods can be applied even before the immediate manifestation of stroke, e.g., to individuals who have been identified as a very high risk a few hours before the onset of stroke. Methods for personalised stroke prediction a few hours ahead or even a day ahead have already been developed to identify individuals with a very high risk before stroke manifests. The data used are longitudinal, multi-modal data of environmental variables measured over several days, such as changes in temperature, solar eruptions, pollution, wind direction, geomagnetic storms [107], etc., combined with personal data, such as age, blood pressure, smoking habits, etc. [108,109,110,111,112]. These methods can predict individual stroke with an accuracy of 95% and more than a few hours or a day ahead, after which MRI data can be measured and analysed for high-risk subjects to try to prevent the onset of the event.

In closing, the performance of the deep learning models needs to be improved to replace human effort; however, they can still provide robust, accurate, adaptable solutions to help professionals and patients alike.

## Figures and Tables

**Table 2:** Comprehensive analysis of the review papers.

Previous Studies	Highlights
[22]	Presented the different object recognition techniques that can perform well on lesion segmentation. Thorough performance analysis was performed on the discussed techniques.
[27]	Discussed the different types of stroke and presented the different studies conducted for each stroke type. The study focused on the different imaging modalities and their impact on lesion segmentation. Presented the openly available datasets for lesion segmentation and detection.
[6]	Thoroughly discussed the state-of-the-art lesion segmentation methods. Presented some challenges of using deep learning techniques for stroke lesion segmentation.
[28]	Deep learning techniques for lesion segmentation were discussed in detail. The different factors used to evaluate the performance of the deep learning model regarding lesion segmentation were also discussed.
[29]	A thorough analysis was made of the various pre-processing, segmentation, and classification techniques used for stroke analysis.
[30]	The paper presented many state-of-the-art deep learning techniques and studies.
[31]	Discussed the state-of-the-art deep learning techniques and characterised them based on image modality.

**Table 3:** Overview of the articles included in the literature.

Reference	Input Modalities	Dataset	Pre-Processing	Structure		Loss Function	Performance Metrics		
				Feature Extraction	Segmentation		Dice-Coef	Precision	Recall
[12]	MRI T1	ATLAS v1.2	Not Mentioned	CNN-based encoder with different scales	CNN-based decoder with residual encoder	Combination of binary cross-entropy loss and dice coefficient loss	0.6627	0.6942	0.664
[64]	MRI T1	ATLAS v1.2	Not Mentioned	CNN-based encoder with 3D convolutional layer	CNN-based decoder with dimension transformation block	Combination of focal loss and dice coefficient loss	0.7231	0.6331	0.5243
[76]	MRI T1	ATLAS v1.2	Not Mentioned	Depth-wise convolution-based encoder with a feature similarity module	Depth-wise CNN-based decoder	Sum of dice loss and cross-entropy loss	0.4867	0.6	0.4752
[33]	MRI T1, T2, FLAIR, DWI	ISLES 2015	Symmetric modality; augmentation using image registration	CNN-based encoder with residual connections	CNN-based decoder	Focal loss	SISS: 0.59 SPES: 0.84	Not Mentioned	Not Mentioned
[65]	MRI DWI, MTT, CBV, CTP	ISLES (version not mentioned)	Normalization (min-max normalization)	CNN-based encoder with densely connected paths for each modality	CNN-based decoder	Not Mentioned	0.635	Not Mentioned	Not Mentioned
[41]	MRI T1	ATLAS v1.2	Normalized to MNI-152 space	CNN-based encoder with Multi-scale Deep Fusion unit	CNN-based decoder	Dice loss	0.6875	Not Mentioned	Not Mentioned
[42]	MRI T1, WI, ADC, DWI, and FLAIR	ATLAS v1.2 and ISLES 2022	Transfer learning; normalized to MNI-152 space	Two encoders: CNN-based capturing local features and transformer-based for capturing global features with Boundary Deformation Module	CNN-based decoder with Boundary Constraint Module	Multi-task learning loss	ATLAS: 0.6167 ISLES 2022: 0.856	ATLAS: 0.6286 ISLES 2022: 0.8834	ATLAS: 0.6868 ISLES 2022: 0.8539
[71]	CT CBV, CBF, $T_{max}$, MTT	ISLES 2018	Not Mentioned	CNN-based encoder with DenseNet-inspired blocks for each layer	CNN-based decoder	Combination of dice coefficient and cross-entropy function	0.44	0.54	0.44
[56]	CT CBV, CBF, $T_{max}$, MTT	ISLES 2018	Bias correction; Standardization (z-score normalization)	CNN-based encoder with dilated convolutions	CNN-based decoder	Not Mentioned	0.37	0.44	0.44
[77]	MRI T1	ATLAS v1.2	Transfer learning for transformer layer	CNN-based encoder with patch partition block and attention-based transformer	CNN-based decoder	Combination dice loss and weighted binary cross-entropy loss	0.6119	0.633	0.6765
[73]	CT CBV, CBF, $T_{max}$, MTT	ISLES 2018	Not Mentioned	CNN-based encoder with localized and dilated convolution layers	CNN-based decoder	Intersection over union	0.58	0.68	0.6
[55]	CT CBV, CBF, $T_{max}$, MTT	ISLES 2018	Bias correction; normalization	CNN-based encoder with residual inception block and dense blocks	CNN-based decoder with residual inception block and dense blocks	Combination dice loss and binary cross-entropy loss	0.82	0.77	0.9
[54]	MRI	ISLES 2015	Skull stripping; Standardization (z-score normalization); transfer learning	CNN-based encoder	CNN-based decoder	Dice loss	0.7	Not Mentioned	Not Mentioned
[78]	MRI DWI, FLAIR, T1, T2	ISLES 2015	Not Mentioned	CNN-based encoder with Multi-Res Attention Block	CNN-based decoder with pixel majority class voting	Combination of dice coefficient and categorical cross-entropy loss	0.7752	0.7513	Not Mentioned
[34]	CT CBV, CBF, $T_{max}$, MTT	ISLES 2018	Intensity clipping; Bilinear interpolation; Standardization (z-score normalization)	CNN-based encoder with Multi-Res Blocks	CNN-based decoder with CNN shortcuts	Binary Cross-Entropy Loss	0.68	Not Mentioned	Not Mentioned
[40]	MRI T1	ATLAS v1.2	Normalized to MNI-152 space	Primary and auxiliary CNN-based encoders	Primary and auxiliary CNN-based decoders	WBCE-Tversky loss for primary encoder; tolerance loss for auxiliary encoder	0.592	0.656	0.599
[35]	MRI T1	ATLAS v1.2	Bilinear interpolation	CNN-based encoders with Cross-Spatial Attention Module	CNN-based decoder	Combination dice loss and binary cross-entropy loss	0.5561	0.6368	0.5817
[66]	CT CBV, CBF, $T_{max}$, MTT	ISLES 2018	Normalization (percentile clipping)	Temporal Sampling, Temporal MIP, and CNN-based encoder	CNN-based decoder	Combination of weighted cross-entropy and hardness-aware generalized dice loss	0.51	0.55	0.55
[13]	MRI T1	ATLAS v1.2	Not Mentioned	CNN-based model inspired from visual cortex		Combination of EML loss (proposed in [64]) with binary cross-entropy loss	0.8449	0.5349	Not Mentioned
[79]	MRI DWI, ADC, T2W1	Training: Internal dataset Evaluation: ISLES 2015	Standardization (z-score normalization)	ResNet-inspired encoder	Global convolution network (GCN)-based decoder	Negative dice coefficient	0.55	0.61	0.6
[52]	CT CBV, CBF, $T_{max}$, MTT	Internal dataset	Skull stripping; Standardization (z-score normalization); transfer learning	DenseNet-based encoder	CNN-based decoder	Combination of weighted cross-entropy and dice loss	0.43	0.53	0.45
[80]	MRI eADC, DWI	Internal dataset	Not Mentioned	Transformer-based encoder	MoE-based decoder	Intersection over union	0.88	Not Mentioned	Not Mentioned
[68]	MRI eADC, DWI	Internal dataset	Not Mentioned	Lambda layers-based encoder	CNN-based decoder	Binary cross-entropy loss	0.8651	0.8939	0.8176
[53]	MRI DWI	Internal dataset	Skull stripping; Standardization (z-score normalization); transfer learning	DeepMedic-based semi-supervised student–teacher model		Combination of soft dice loss (used for calculating loss of unannotated data) and cross-entropy loss (calculated for annotated data)	0.6676	Not Mentioned	Not Mentioned
[86]	MRI DWI, ADC	Internal dataset	Standardization (z-score normalization)	Semi-supervised VVG-16-based model		Binary cross-entropy loss	0.699	0.852	0.923
[89]	CT CBF, DPWI	ISLES 2018	Not Mentioned	GAN with U-Net-based generator and FCN-based discriminator		Not Mentioned	0.39	0.55	0.36
[91]	MRI T1	ATLAS v1.2	Not Mentioned	GAN using U-Net-based segmentation module		Segmentation model: dice loss; discriminator: hybrid Loss function	0.617	0.63	Not Mentioned
[90]	Internal dataset: MRI eADC, DWI; ISLES 2022: MRI DWI, ADC, FLAIR	Internal dataset and ISLES 2022	Not Mentioned	GAN with Patcher-based generator and FCN-based discriminator		Adversarial loss and cross-entropy loss	0.8362	Not Mentioned	Not Mentioned

## Supplementary Materials

The following supporting information can be downloaded at https://www.mdpi.com/article/10.3390/bioengineering11010086/s1. Table S1: Quality Assessment of all the articles included in the literature.
