# Supplementary material for: Stroke Lesion Segmentation and Deep Learning: A Comprehensive Review
Source: Bioengineering (Basel). 2024 Jan 17;11(1):86. doi: 10.3390/bioengineering11010086 (PMC10813717; doi:10.3390/bioengineering11010086)
Supplement: Supplementary file 1 [file bioengineering-11-00086-s001.zip › bioengineering-2796461-supplementary.pdf]

Table S1. Quality Assessment of all the articles included in the literature.

Reference	Q1	Q2	Q3	Q4	Q5	Q6	Q7	Q8	Q9	Q10	Total
[12]	1	1	1	1	1	1	1	0	0	0	7
[64]	1	1	1	1	1	1	1	0	0	0	7
[76]	1	1	1	0	1	0	1	0	0	1	6
[33]	1	1	1	1	1	0	0	1	0	1	7
[65]	1	1	1	0	1	1	0	1	0	0	6
[41]	1	1	1	0	1	1	1	0	0	0	6
[42]	1	1	1	0	1	0	1	1	0	0	6
[71]	1	1	1	1	1	1	1	1	0	0	8
[56]	0	1	1	1	1	1	0	0	0	1	6
[77]	1	1	1	0	1	1	1	0	0	0	6
[73]	1	1	1	0	1	1	1	0	0	0	6
[55]	1	1	1	1	1	1	1	0	0	1	8
[54]	1	1	1	1	1	0	0	0	0	0	5
[78]	1	0	1	1	1	1	1	0	0	0	6
[34]	1	1	1	1	1	1	1	1	0	0	8
[40]	1	1	1	1	1	1	1	0	0	0	7
[35]	1	1	1	1	1	1	1	0	0	0	7
[66]	1	1	1	1	1	1	1	1	0	0	8
[13]	0	1	0	1	1	0	1	0	0	0	4
[79]	1	1	1	1	1	1	1	1	0	0	8
[52]	0	0	0	1	0	1	1	0	0	0	3
[80]	1	1	0	0	1	0	1	0	0	1	5
[68]	1	1	0	0	1	1	1	0	0	1	6

[53]	1	1	0	1	1	1	1	0	0	0	6
[86]	1	1	0	0	1	1	1	0	0	0	5
[89]	0	1	1	0	1	1	0	0	0	0	4
[91]	1	1	1	0	1	0	1	0	0	0	5
[90]	1	1	0	0	1	0	1	0	0	0	4
Average	0.86	0.93	0.75	0.57	0.96	0.71	0.82	0.25	0	0.21	6.07

References:

[12] Zhang, X.; Xu, H.; Liu, Y.; Liao, J.; Cai, G.; Su, J.; Song, Y. A Multiple Encoders Network for Stroke Lesion Segmentation. In Proceedings of the Chinese Conference on Pattern Recognition and Computer Vision (PRCV), Virtual Conference, 29 October–1 November 2021; Springer International Publishing: Cham, Switzerland, 2021; 524–535.

[13] Li, C. Stroke Lesion Segmentation with Visual Cortex Anatomy Alike Neural Nets. *arXiv* **2021**, arXiv:2105.06544.

[33] Clèrigues, A.; Valverde, S.; Bernal, J.; Freixenet, J.; Oliver, A.; Lladó, X. Acute and sub-acute stroke lesion segmentation from multimodal MRI. *Comput. Methods Programs Biomed*. **2020**, *194*, 105521.

[34] Soltanpour, M.; Greiner, R.; Boulanger, P.; Buck, B. Improvement of automatic ischemic stroke lesion segmentation in CT perfusion maps using a learned deep neural network. *Comput. Biol. Med.* **2021**, *137*, 104849.

[35] Sheng, M.; Xu, W.; Yang, J.; Chen, Z. Cross-Attention and Deep Supervision UNet for Lesion Segmentation of Chronic Stroke. *Front. Neurosci.* **2022**, *16*, 836412.

[40] Hui, H.; Zhang, X.; Wu, Z.; Li, F. Dual-path attention compensation U-Net for stroke lesion segmentation. *Comput. Intell. Neurosci.* **2021**, *2021*, 7552185.

[41] Liu, X.; Yang, H.; Qi, K.; Dong, P.; Liu, Q.; Liu, X.; Wang, R.; Wang, S. MSDF-Net: Multi-scale deep fusion network for stroke lesion segmentation. *IEEE Access*, **2019**, 7, 178486–178495.

[42] Wu, Z.; Zhang, X.; Li, F.; Wang, S.; Huang, L.; Li, J. W-Net: A boundary-enhanced segmentation network for stroke lesions. *Expert Syst. Appl.* **2023**, *230*, 120637.

[52] Anand, V.K.; Khened, M.; Alex, V.; Krishnamurthi, G. Fully automatic segmentation for ischemic stroke using CT perfusion maps. In Brainlesion: Glioma, Multiple Sclerosis, Stroke and Traumatic Brain Injuries: 4th International Workshop, BrainLes 2018, Held in Conjunction with MICCAI 2018, Granada, Spain, 16 September 2018; Revised Selected Papers, Part I; Springer International Publishing; pp. 328–334.

[53] Cui, W.; Liu, Y.; Li, Y.; Guo, M.; Li, Y.; Li, X.; Wang, T.; Zeng, X.; Ye, C. Semi-supervised brain lesion segmentation with an adapted mean teacher model. In *Information Processing in Medical Imaging, Proceedings of the 26th International Conference, IPMI 2019, Hong Kong, China, 2–7 June 2019*; Proceedings 26; Springer International Publishing; pp. 554–565.

[54] Karthik, R.; Gupta, U.; Jha, A.; Rajalakshmi, R.; Menaka, R. A deep supervised approach for ischemic lesion segmentation from multimodal MRI using Fully Convolutional Network. *Appl. Soft Comput.* **2019**, *84*, 105685.

[55] Ahmad, P.; Jin, H.; Alroobaea, R.; Qamar, S.; Zheng, R.; Alnajjar, F.; Aboudi, F. MH UNet: A multi-scale hierarchical based architecture for medical image segmentation. *IEEE Access* **2021**, *9*, 148384–148408.

[56] Tureckova, A.; Rodríguez-Sánchez, A.J. ISLES challenge: U-shaped convolutional neural network with dilated convolution for 3D stroke lesion segmentation. In *Brainlesion: Glioma, Multiple Sclerosis, Stroke and Traumatic Brain Injuries, Proceedings of the 4th International Workshop, BrainLes 2018, Held in Conjunction with MICCAI 2018, Granada, Spain, 16 September 2018*; Revised Selected Papers, Part I; Springer International Publishing; pp. 319–327.

[64] Zhou, Y.; Huang, W.; Dong, P.; Xia, Y.; Wang, S. D-UNet: A dimension-fusion U shape network for chronic stroke lesion segmentation. *IEEE/ACM Trans. Comput. Biol. Bioinform.* **2019**, *18*, 940–950.

[65] Dolz, J.; Ben Ayed, I.; Desrosiers, C. Dense multi-path U-Net for ischemic stroke lesion segmentation in multiple image modalities. In Brainlesion: Glioma, Multiple Sclerosis, Stroke and Traumatic Brain Injuries, Proceedings of the 4th International Workshop, BrainLes 2018, Held in Conjunction with MICCAI 2018, Granada, Spain, 16 September 2018; Revised Selected Papers, Part I; Springer International Publishing; pp. 271–282.

[66] Wang, G.; Song, T.; Dong, Q.; Cui, M.; Huang, N.; Zhang, S. Automatic ischemic stroke lesion segmentation from computed tomography perfusion images by image synthesis and attention-based deep neural networks. *Med. Image Anal.* **2020**, *65*, 101787.

[68] Ou, Y.; Yuan, Y.; Huang, X.; Wong, K.; Volpi, J.; Wang, J.Z.; Wong, S.T. Lambdaunet: 2.5D stroke lesion segmentation of diffusion-weighted MR images. In *Medical Image Computing and Computer Assisted Intervention—MICCAI 2021, Proceedings of the 24th International Conference, Strasbourg, France, 27 September–1 October 2021*; Proceedings, Part I; Springer International Publishing; pp. 731–741.

[71] Liu, L.; Yang, S.; Meng, L.; Li, M.; Wang, J. Multi-scale deep convolutional neural network for stroke lesions segmentation on CT images. In *Brainlesion: Glioma, Multiple Sclerosis, Stroke and Traumatic Brain Injuries, Proceedings of the 4th International Workshop, BrainLes 2018, Held in Conjunction with MICCAI 2018, Granada, Spain, 16 September 2018*; Revised Selected Papers, Part I; Springer International Publishing; pp. 283–291.

[73] Omarov, B.; Tursynova, A.; Postolache, O.; Gamry, K.; Batyrbekov, A.; Aldeshov, S., Azhibekova, Z.; Nurtas, M.; Aliyeva, A.; Shiyapov, K. Modified UNet Model for Brain Stroke Lesion Segmentation on Computed Tomography Images. *Comput. Mater. Contin.* **2022**, *71*, 4701–4717.

[76] Qi, K.; Yang, H.; Li, C.; Liu, Z.; Wang, M.; Liu, Q.; Wang, S. X-net: Brain stroke lesion segmentation based on depthwise separable convolution and long-range dependencies. In Medical Image Computing and Computer Assisted Intervention—Proceedings of the MICCAI 2019: 22nd International Conference, Shenzhen, China, 13–17 October 2019; Proceedings, Part III; Springer International Publishing; pp. 247–255.

[77] Wu, Z.; Zhang, X.; Li, F.; Wang, S.; Huang, L. Multi-scale long-range interactive and regional attention network for stroke lesion segmentation. *Comput. Electr. Eng.* **2022**, *103*, 108345.

[78] Karthik, R.; Menaka, R.; Hariharan, M.; Won, D. Ischemic lesion segmentation using ensemble of multi-scale region aligned CNN. Comput. Methods Programs Biomed. 2021, 200, 105831.

[79] Liu, Z.; Cao, C.; Ding, S.; Liu, Z.; Han, T.; Liu, S. Towards clinical diagnosis: Automated stroke lesion segmentation on multi-spectral MR image using convolutional neural network. *IEEE Access* **2018**, *6*, 57006–57016.

[80] Ou, Y.; Yuan, Y.; Huang, X.; Wong, S.T.; Volpi, J.; Wang, J.Z.; Wong, K. Patcher: Patch transformers with mixture of experts for precise medical image segmentation. In *Proceedings of the International Conference on Medical Image Computing and Computer-Assisted Intervention, Singapore, 18–22 September 2022*; Springer Nature Switzerland: Cham, Switzerland, 2022; pp. 475–484.

[86] Zhao, B.; Liu, Z.; Liu, G.; Cao, C.; Jin, S.; Wu, H.; Ding, S. Deep learning-based acute ischemic stroke lesion segmentation method on multimodal MR images using a few fully labeled subjects. *Comput. Math. Methods Med.* **2021**, *2021*, 3628179.

[89] Islam, M.; Vaidyanathan, N.R.; Jose, V.J.M.; Ren, H. Ischemic stroke lesion segmentation using adversarial learning. In Brainlesion: Glioma, Multiple Sclerosis, Stroke and Traumatic Brain Injuries, Proceedings of the 4th International Workshop, BrainLes 2018, Held in Conjunction with MICCAI 2018, Granada, Spain, 16 September 2018; Revised Selected Papers, Part I; Springer International Publishing, 292–300.

[90] O u, Y.; Huang, S.X.; Wong, K.K.; Cummock, J.; Volpi, J.; Wang, J.Z.; Wong, S.T. BBox-Guided Segmentor: Leveraging expert knowledge for accurate stroke lesion segmentation using weakly supervised bounding box prior. *Comput. Med. Imaging Graph.* **2023**, *107*, 102236.

[91] Wang, S.; Chen, Z.; You, S.; Wang, B.; Shen, Y.; Lei, B. Brain stroke lesion segmentation using consistent perception generative adversarial network. *Neural Comput. Appl.* **2022**, *34*, 8657–8669
